# Hepatocellular Carcinoma Arising From Well‐Differentiated Hepatocellular Neoplasm of Uncertain Malignant Potential in a Patient With Abernethy Malformation

**DOI:** 10.1155/cris/5595846

**Published:** 2026-09-16

**Authors:** Keiichi Kinowaki, Jacqueline E. Birkness-Gartman, Ihab Kamel, Linda Chu, Robert A. Anders, Christopher R. Shubert, Kiyoko Oshima

**Affiliations:** ^1^ Department of Pathology, Johns Hopkins University School of Medicine, Baltimore, Maryland, USA, jhu.edu; ^2^ Department of Pathology, Toranomon Hospital, Tokyo, Japan, toranomon.gr.jp; ^3^ Okinaka Memorial Institute for Medical Research, Tokyo, Japan; ^4^ Department of Radiology, Johns Hopkins University School of Medicine, Baltimore, Maryland, USA, jhu.edu; ^5^ Department of Surgery, Johns Hopkins University School of Medicine, Baltimore, Maryland, USA, jhu.edu; ^6^ Department of Pathology, University of Pittsburgh Medical Center, Pittsburgh, Pennsylvania, USA, upmc.com

**Keywords:** Abernethy malformation, congenital extrahepatic mesocaval shunt (CEMS), hepatocellular neoplasm of uncertain malignant potential (HUMP), transformation

## Abstract

Abernethy malformation is a rare congenital extrahepatic mesocaval shunt (CEMS) in which mesenteric venous blood bypasses the liver completely (type I) or partially (type II) and drains directly into the vena cava. In complete CEMS (type I), the intrahepatic portal vein is absent. The resulting lack of portal flow is associated with the development of hepatic nodules, such as focal nodular hyperplasia (FNH), hepatocellular adenoma (HCA), and hepatocellular carcinoma (HCC). These lesions have a higher risk of malignant transformation than conventional adenomas. The term hepatocellular neoplasm of uncertain malignant potential (HUMP) has been proposed for borderline lesions between HCA and well‐differentiated HCC, diagnosed based on atypical histological, genetic, or clinical features suggesting a possible malignant potential. However, the exact risk of malignant transformation is unknown. We present the case of a 32‐year‐old man with Type I Abernethy malformation who developed malignant transformation of a HUMP over a 15‐year period. Serial hepatic resections performed at other institutions demonstrated histological progression from HUMP to HCC with subsequent lung metastasis. This gradual transformation has been documented in several surgical specimens. This case highlights the need for careful recognition of HUMP in patients with Abernethy malformation, as timely liver transplantation may be essential to prevent malignant transformation and achieve curative outcomes.

## 1. Introduction

In 2014, Bedossa et al. [1] first proposed the concept of a well‐differentiated hepatocellular neoplasm of uncertain malignant potential (HUMP). This term describes borderline hepatocellular lesions that lie between hepatocellular adenoma (HCA) and well‐differentiated hepatocellular carcinoma (HCC), and the diagnosis relies on atypical histological, genetic, or clinical features [1]. Contemporary practice increasingly integrates molecular subclassification, particularly β‐catenin and glutamine synthetase immunohistochemistry, to refine risk stratification and guide management decisions [2, 3]. Although the HUMP criteria acknowledge an association with malignancy, the true risk of malignant transformation remains unclear.

Abernethy malformation is a rare congenital extrahepatic mesocaval shunt (CEMS) in which mesenteric venous blood is diverted, either completely (type I) or partially (type II), into the systemic circulation. Type I Abernethy malformations lack an intrahepatic portal vein; therefore, the liver predominantly depends on the arterial blood supply. Hepatic nodules, such as focal nodular hyperplasia (FNH), HCA, and HCC, occur in 40%–65% of affected patients [4, 5].

Herein, we report a case of type I Abernethy malformation complicated by HUMP that underwent malignant transformation. During the 18 years of clinical follow‐up, histological progression from HUMP to HCC was observed. This case provides rare and valuable insights into the long‐term malignant potential and evolution of HUMP, contributing to our understanding of its natural history. Furthermore, this underscores the importance of timely surgical intervention as appropriate resection may prevent malignant transformation.

## 2. Case Presentation

A 32‐year‐old man with a history of complete CEMS (type I Abernethy malformation), absence of the intrahepatic portal vein, and multiple liver masses presented to our institution. The patient was first diagnosed at the age of 17 years at another institution with a vascular malformation and a hepatic adenoma. Imaging surveillance was initiated for the patient. Gadoxetic acid‐enhanced MRI performed prior to the first surgical intervention demonstrated a heterogeneous T2 hyperintense mass in the right hepatic lobe, initially interpreted as FNH, and a mildly heterogeneous T2 isointense mass in the left hepatic lobe showing heterogeneous hypoenhancement in the arterial, venous, and hepatobiliary phases, imaging features that overlap with those of HCA (Figure S1). At the age of 20 years, the patient underwent his first liver resection. Pathological examination at an external facility indicated HCA with positive surgical margins.

Subsequent magnetic resonance imaging revealed tumor regrowth, and a second liver resection was performed when the patient was 26 years old. The lesion was again diagnosed as an HCA; however, residual or recurrent adenomas continued to enlarge. At 28 years of age, the patient underwent multiple microwave ablation procedures. He was offered an evaluation for liver transplantation; however, he declined because of the diagnosis of an adenoma.

**Figure 1:**
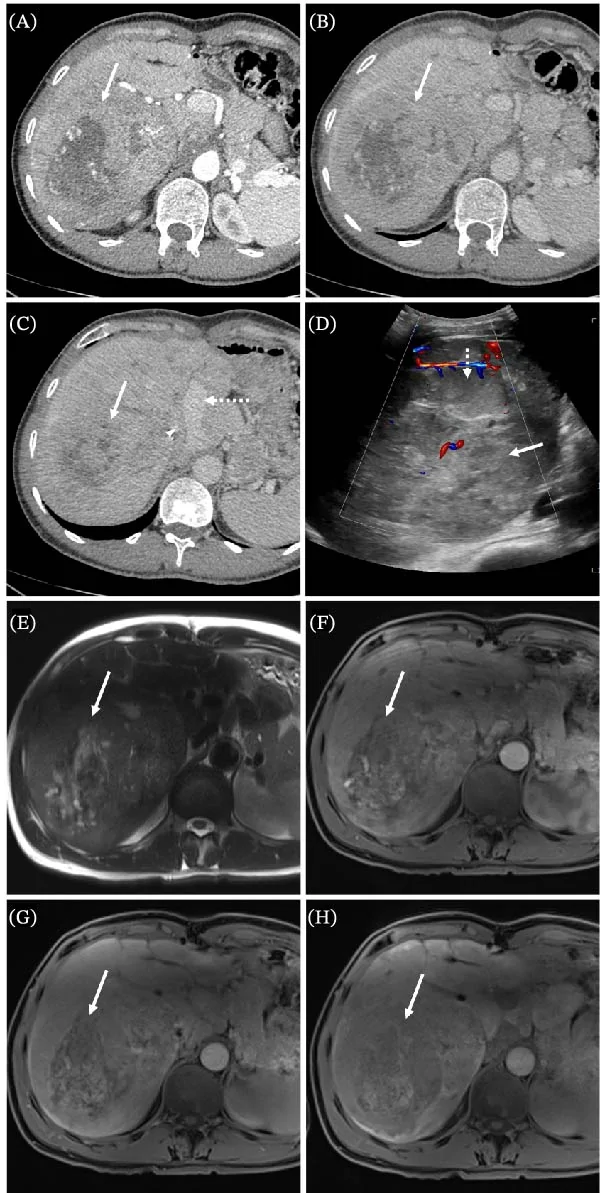
(A–C) Axial contrast‐enhanced CT scan of the abdomen in the arterial phase (A) and portal venous phase (B, C) demonstrates a large heterogeneously enhancing mass in the right hepatic lobe (arrow) with central necrosis in both the arterial and portal venous phases. A portocaval shunt was also observed (arrowhead) (C). (D) Grayscale and color Doppler ultrasound image of the right hepatic lobe showed a large heterogeneous mass (arrow) with prominent internal vascularity and feeding vessels (dotted arrow). (E–H) The axial T2‐weighted MR image (E) shows a heterogeneous mass with predominantly intermediate signal intensity and central T2 hyperintensity corresponding to areas of necrosis. Axial T1‐weighted MR images obtained with gadoxetic acid in the arterial phase (F), portal venous phase (G), and 20‐min hepatobiliary phase (H) show a heterogeneously enhancing mass with areas of arterial phase hyperenhancement and washout in the portal venous phase, and a hypointense signal relative to the background liver in the hepatobiliary phase.

At the age of 33 years, the patient presented to our institution for a second opinion. Contrast‐enhanced CT in the arterial and portal venous phases revealed a large, heterogeneously enhancing mass in the right hepatic lobe with central necrosis and a portocaval shunt. Grayscale and color Doppler ultrasonography revealed a heterogeneous mass with prominent internal vascularity and feeding vessels. Gadoxetic acid‐enhanced MRI revealed a mass measuring ~13 cm with arterial phase hyperenhancement, portal venous phase washout, and hepatobiliary phase hypointensity relative to the background liver, findings that were consistent with HCC (Figure 1). His alpha‐fetoprotein (AFP) level was markedly elevated at 2311 ng/mL (upper limit of normal: ≤10.0 ng/mL). Ultrasound‐guided biopsy revealed a well‐differentiated HCC. Given the tumor’s size, number, and elevated AFP level, the patient was deemed ineligible for a total hepatectomy and liver transplantation at that time. Local therapy was offered to attempt tumor downstaging for potential future transplantation.

One month later, the patient underwent liver resection at a different facility. Seven months postoperatively, computed tomography revealed multiple pulmonary nodules that had progressively increased in size. Ten months later, right middle lobe lung resection was performed, and pathological examination confirmed the diagnosis of metastatic HCC. At present, 3 years after lung surgery, the patient remains alive with recurrent HCC in the liver and metastases to the bone and lung. He received radiation therapy, followed by systemic therapy with atezolizumab and bevacizumab.

Histopathological review of the first resection specimen revealed hepatocytes with numerous exposed vessels without accompanying bile ducts. Focal pseudoglandular formations were present, but cytologic atypia was minimal. Reticulin staining demonstrated focal thickening of the hepatic cords. The second resection specimen exhibited an increased frequency of pseudoglandular formations, with reticulin staining showing focal thickening of the hepatic cord. In retrospect, both earlier specimens showed sufficient architectural abnormalities to raise concerns regarding malignant transformation. Based on these features and the fulfillment of the diagnostic criteria [1], the lesion was diagnosed as HUMP.

**Figure 2:**
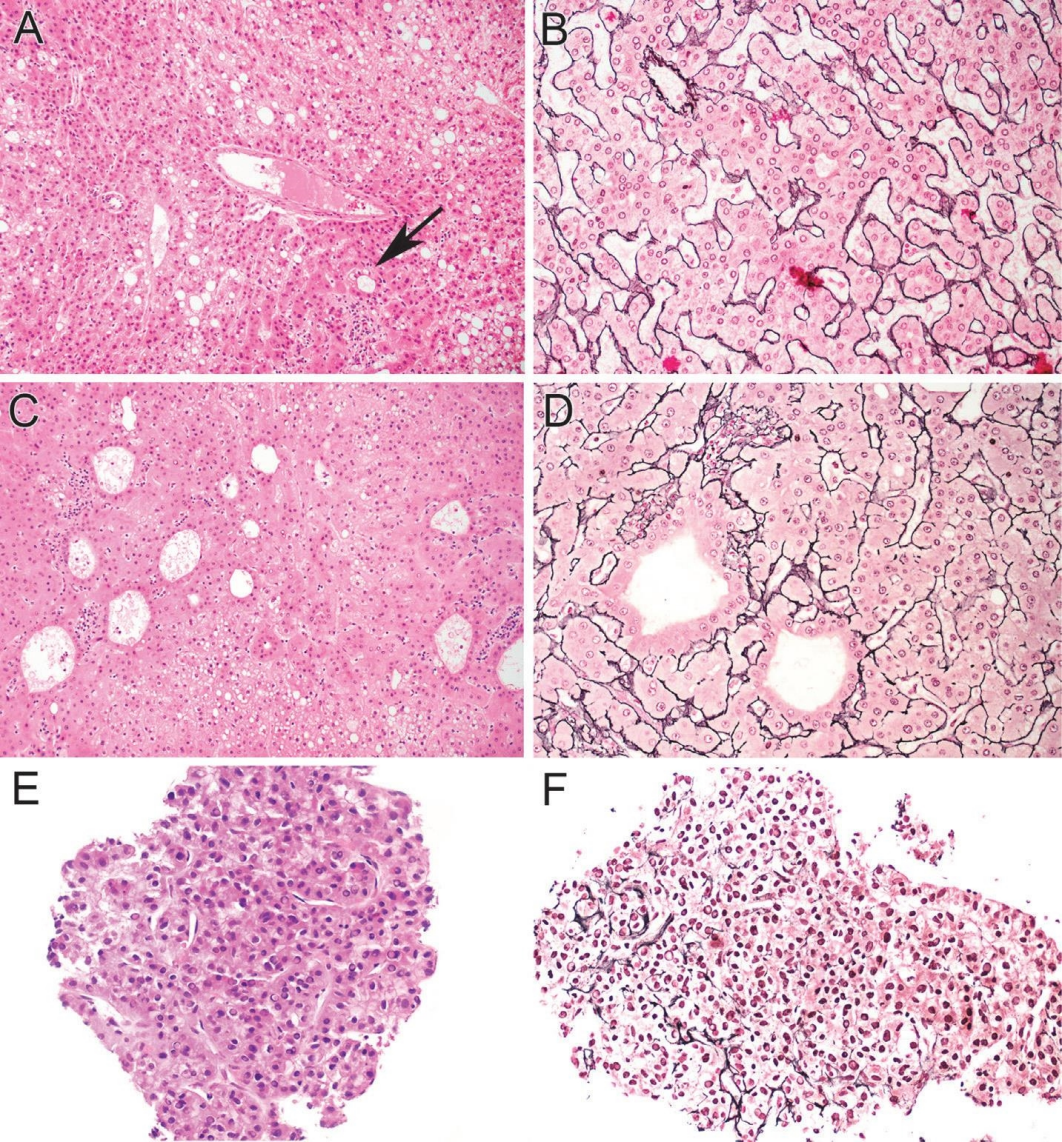
(A) Hematoxylin and eosin (HE) section from the first resection specimen (age 20) showing multiple naked vessels without accompanying bile ducts. Cytological atypia was minimal; however, rare pseudoglandular formation was observed (arrows). (B) Reticulin stain from the first resection specimen (age 20) highlighting focally thickened hepatic cords. (C) Hematoxylin and eosin (HE) staining of the second resection specimen (age 26 years) showing minimal cytological atypia with more frequent pseudoglandular formations. (D) Reticulin stain from the second resection specimen (age 26) highlighting pseudoglandular formations and focally thickened hepatic cords. (E) Hematoxylin and eosin (HE) section from the most recent liver biopsy (age 33) demonstrating cytologic atypia characterized by small‐cell changes. (F) Reticulin stain from the most recent liver biopsy (age 33) showing complete loss of the reticulin network.

In the most recent biopsy, hepatocyte atypia was pronounced with diffuse small‐cell changes. Reticulin loss was extensive, and glutamine synthetase demonstrated diffuse expression, confirming HCC. Immunostaining for β‐catenin was performed on all specimens and showed membranous (nonnuclear) labeling (Figure 2).

## 3. Discussion

Abernethy malformation is a rare congenital vascular anomaly in which mesenteric venous blood partially or completely drains into the systemic circulation [4, 6]. First described by Abernethy in 1793, this condition may present with various complications, such as hyperbilirubinemia, hyperammonemia, hepatic encephalopathy, pulmonary hypertension, and hepatopulmonary syndrome, as most or all intestinal and splenic venous blood bypasses the liver. In type I Abernethy malformation, the absence of portal flow predisposes the liver to nodular formation and malignant transformation. Over half of individuals with CEMS develop benign or malignant liver tumors, and type I Abernethy malformation can be associated with multiple tumor types [7]. Liver lesions are reported in 40%–65% of patients with type I disease [4, 5]. FNH, nodular regenerative hyperplasia, adenoma, and hemangioma can arise due to altered hepatic hemodynamics, increased arterial flow, lack of portal flow, and elevated circulating liver growth factors.

Abernethy malformations have been linked to hepatoblastoma and HCC, even at a young age and in the absence of cirrhosis [4, 6]. Malignant transformation of pre‐existing benign tumors appears to be more common than the de novo development of primary malignant tumors. Adenomas associated with vascular disorders have a distinct molecular pathogenesis and prognosis compared to conventional adenomas, with a higher risk of malignant transformation [8]. Recent molecular studies have demonstrated that FNH‐like nodules and adenomas in vascular disorders frequently show β‐catenin activation [3, 9], providing a molecular basis for the increased malignant potential in this condition.

Malignant transformation occurs in HCA, with the risk varying according to the molecular subtype. β‐catenin‐activated HCAs carry the highest risk [10, 11]. Recent molecular studies have demonstrated particularly high frequencies of CTNNB1 variants in hepatic tumors arising from congenital portosystemic shunts [9], with studies reporting transformation rates of 20%–30% in CTNNB1 exon 3‐mutated lesions [11]. Among studies on borderline lesions, well‐differentiated hepatocellular neoplasms with localized atypia that are insufficient to meet the HCC diagnostic criteria have been of particular interest. Targeted next‐generation sequencing has revealed frequent CTNNB1 variants and other molecular alterations in hepatic tumors arising from congenital portosystemic shunts [9, 10], confirming β‐catenin pathway activation as a key driver of the malignant potential of these lesions. In 2014, Bedossa et al. [1] introduced the term HUMP for lesions that cannot be definitively classified as HCA or HCC. The HUMP terminology was introduced to convey to the clinical team that these lesions carry a risk of transformation significant enough to warrant clinical follow‐up or intervention. Other authors have referred to these as “atypical hepatocellular neoplasms” or “well‐differentiated hepatocellular lesions.”

The diagnosis of HUMP is based on atypical histological, genetic, or clinical features. The histologic criteria include focal reticulin loss, focal cytologic atypia (such as small‐cell change or nuclear atypia), or focal architectural atypia, such as pseudoglandular formation involving <5% of the tumor. β‐Catenin mutations are considered atypical. Clinically, HCAs occurring in males, older women (>50 years), younger girls (<15 years), and patients using anabolic steroids are classified as atypical [1]. Lesions associated with glycogen storage disease, tyrosinemia, galactosemia, and congenital hepatic fibrosis have also been proposed to fall under the HUMP category [12].

An important consideration in this case is whether the HCC arose through malignant transformation of a pre‐existing HUMP or represented a de novo secondary malignancy induced by environmental factors such as chronic ischemia, hypoperfusion, or inflammatory stress associated with the portosystemic shunt. Several lines of evidence support the interpretation of progressive malignant transformation rather than secondary carcinogenesis.

Histopathological review of the first (20 years old) and second (26 years old) resection specimens demonstrated architectural features characteristic of HUMP, including focal pseudoglandular formation and focal thickening of hepatic cords on reticulin staining. These findings suggest that the lesion harbored atypical features at the initial presentation rather than arising de novo. Across the three time points examined, the lesion demonstrated a stepwise increase in architectural complex and cytologic atypia, culminating in pronounced small‐cell changes and reticulin loss in the most recent biopsy. The gradual histological evolution supports a continuum of transformation rather than the abrupt appearance of an independent malignancy.

Recent molecular studies have demonstrated that hepatic tumors arising in congenital portosystemic shunts frequently harbor CTNNB1 mutations across both benign and malignant lesions [9], suggesting a shared molecular pathogenesis rather than independent tumor development. While we acknowledge that molecular testing was not performed in our case, which is a significant limitation, the histological progression documented through serial resection provides compelling evidence for the malignant transformation of a single evolving lesion. While secondary carcinogenesis driven by chronic environmental stress cannot be entirely excluded, the chronological pathological changes observed in our case are more consistent with the stepwise malignant transformation of HUMP into HCC.

Distinguishing adenomas from HCC based solely on histology is challenging. Although the HUMP definition allows focal atypia involving < 5% of the tumor [1], this cutoff is arbitrary, and inter‐observer variation exists even among expert liver pathologists. In particular, the absence of nuclear β‐catenin staining on immunohistochemistry does not exclude CTNNB1 mutations as mutations outside exon 3 or those with low expression levels may not be detectable using standard staining methods [11]. Therefore, additional molecular tests are required to better assess the malignant potential. Importantly, such testing is relevant not only for pathological subclassification but may also provide clinically actionable information, including guidance on the timing of surgery or consideration for transplantation. Pulmonary metastases developed 7 months after the biopsy confirmed HCC. Liver transplantation offers the potential for a cure in patients with Abernethy malformation and adenomas or early‐stage HCC, in contrast to repeated hepatic resections. In our case, molecular analyses were not performed, which is a limitation of this study. Nevertheless, available evidence suggests that the HUMP arising from adenomas associated with underlying vascular abnormalities was identified and that this lesion gradually transformed into HCC. From a clinical perspective, genetic testing can provide prognostic information and influence the timing of surgical intervention, underscoring its importance beyond pathological categorization of tumors.

Overall, our observations underscore the importance of vigilant surveillance and timely evaluation of liver transplantation in patients with vascular anomalies, such as Abernethy malformation, given their increased risk of malignant transformation. This case illustrates HUMP in a patient with type I Abernethy malformation who underwent malignant transformation to HCC after long‐term observation.

While a recent case report described benign‐to‐malignant hepatic lesion transformation in Abernethy malformation, that case demonstrated concurrent FNH and HCC in the explanted liver without documenting the progressive transformation of a single lesion [13]. In contrast, our case represents the first documentation of the natural history of a single hepatocellular lesion progressing from HUMP to HCC through serial resections over an extended follow‐up period.

Patients with this vascular anomaly appear to have a higher risk of malignant transformation than those with conventional HCA. Even minimal histological atypia in this setting should be interpreted with caution as it may indicate the progression to HCC. Early evaluation for liver transplantation, particularly before the development of advanced disease or extrahepatic metastasis, may offer the best chance of cure.

## Author Contributions

Conceptualization: Keiichi Kinowaki and Kiyoko Oshima. Acquisition of clinical data: Keiichi Kinowaki, Ihab Kamel, Linda Chu, Christopher R. Shubert, and Kiyoko Oshima. Drafting or revising the work: Keiichi Kinowaki, Jacqueline E. Birkness‐Gartman, Ihab Kamel, Linda Chu, Robert A. Anders, Christopher R. Shubert, and Kiyoko Oshima. Supervision: Kiyoko Oshima.

## Funding

The authors had no source of funding or financial support, except for themselves.

## Ethics Statement

This case report was exempted from clinical approval by our institution as it reports a single case that emerged during normal clinical practice.

## Consent

Written informed consent was obtained from the participants for the publication of potentially identifiable images or data contained herein.

## Conflicts of Interest

The authors declare no conflicts of interest.

## Supporting Information

Additional supporting information can be found online in the Supporting Information section.

## Supporting information


**Supporting Information** Figure S1: Gadoxetic acid‐enhanced MRI was performed before the first surgical intervention at the age of 20 years. (A) Axial T2‐weighted MR image showed a heterogeneous T2 hyperintense mass in the right hepatic lobe (arrow) and a mildly heterogeneous T2 isointense mass in the left hepatic lobe (dotted arrow). (B–D) Axial T1‐weighted MR images obtained using gadoxetic acid in the arterial (B), portal venous (C), and 10‐min hepatobiliary phases (D) show arterial phase hyperenhancement and venous and hepatobiliary phase isoenhancement of the right hepatic lobe mass. The left hepatic lobe mass showed heterogeneous hypoenhancement during the arterial, venous, and hepatobiliary phases. At the time of the initial imaging, the right hepatic lobe mass was interpreted as focal nodular hyperplasia; on subsequent follow‐up, it enlarged and was ultimately diagnosed as hepatocellular carcinoma (Figure 1). The left hepatic lobe mass was retrospectively classified as a hepatocellular neoplasm of uncertain malignant potential (HUMP) upon second opinion review.

## Data Availability

The datasets underlying the findings of this study are available from the corresponding author upon reasonable requests. These data are not publicly available because of patient privacy and ethical considerations.
